# Evaluation of peripheral blood inflammatory biomarkers in sickle cell disease with and without retinopathy

**DOI:** 10.1007/s00417-024-06569-9

**Published:** 2024-07-08

**Authors:** Ömer Özer, Levent Doğan, Zeki Baysal, Hakan Basir, Ali Türker Çıftçı, Pınar Eröz, Emin Serbülent Güçlü

**Affiliations:** 1 Department of Ophthalmology, Niğde Ömer Halisdemir University, Niğde, 51240 Turkey; 2 Clinic of Internal Medicine, Gülnar State Hospital, Mersin, Turkey; 3 Department of Biostatistics and Medical Informatics, Niğde Ömer Halisdemir University, Niğde, Turkey; 4 Clinic of Ophthalmology, Tarsus State Hospital, Mersin, Turkey; 5 Clinic of Ophthalmology, Mersin State Hospital, Mersin, Turkey

**Keywords:** Anemia, Complete blood count, Disease, İnflammation, Retinopathy, Sickle cell

## Abstract

**Background:**

The aim of this study was to evaluate the clinical significance of blood-cell associated inflammation markers in patients with sickle cell disease (SCD) and sickle cell retinopathy (SCR).

**Methods:**

Neutrophil to lymphocyte ratio (NLR), platelet to lymphocyte ratio (PLR), monocyte to lymphocyte ratio (MLR), systemic immune inflammation index (SIII), systemic inflammation response index (SIRI), systemic inflammation modulation index (SIMI) and aggregate systemic inflammation index (AISI) were calculated. This study included 45 healthy controls (Group 1) and 100 SCD (Group 2). Patients in Group 2 were then divided into two groups: without SCR (Group 3) and with SCR (Group 4), and patients with SCR (Group 4) were further divided into two groups: non-proliferative sickle cell retinopathy (NPSCR) (Group 5) and proliferative sickle cell retinopathy (PSCR) (Group 6).

**Results:**

The mean values for NLR, PLR, SIII, SIRI, AISI, and SIMI were significantly higher in Group 2 compared to Group 1 (*p* = 0.011 for NLR, *p* = 0.004 for SIII, and *p* < 0.001 for others). Furthermore, AISI and SIMI parameters demonstrated statistically significant discriminatory power to distinguish Group 5 from Group 6 (*p* = 0.0016 and *p* = 0.0006, respectively).

**Conclusion:**

Given the critical role of inflammatory mechanisms in the pathogenesis of SCD and its related complications, the assessment of blood-cell-associated inflammatory markers may present a pragmatic and advantageous approach to the clinical oversight and therapeutic intervention of SCD.

## Key messages

***What is known***

• Due to inflammation and local hypoxia, sickle-shaped red blood cells adhere to capillary endothelial cells.

• This leads to decreased vascular flow, vaso-occlusion and microvascular complications.

***What is new***

• Sickle cell patients have increased inflammatory biomarkers compared to healthy.

• Inflammatory biomarkers are increased in sickle cell retinopathy compared to without retinopathy.

• SIMI and AISI are increased in proliferative sickle cell retinopathy.

## Introduction

Sickle cell disease (SCD) is a genetic disorder caused by a mutation in the β-globin gene. This mutation results in the production of hemoglobin S (HbS), which polymerizes upon red blood cell (RBC) deoxygenation. Polymerized HbS causes the RBCs to assume a rigid, sickle-like shape. These sickle-shaped RBCs are highly fragile and susceptible to hemolysis, leading to a shortened lifespan. The imbalance between production and destruction of RBCs results in anemia, the hallmark of SCD [1].

SCD patients suffer from various complications due to this pathophysiological alteration, affecting multiple organs including the lungs, heart, kidneys, brain, skin, bones and eyes. While the molecular defect underlying the disease is well understood, the diverse range of acute and/or chronic complications observed in SCD patients remains a significant challenge in the management of the disease [2].

Complications associated with SCD include painful vaso-occlusive crises, cardiovascular complications, nephropathy, priapism, acute hematogenous osteomyelitis and retinopathy [3–8]. Sickle cell retinopathy (SCR) arises from occlusion in the retinal microcirculation. Reduced deformability of RBCs leads to diminished blood flow in retinal precapillary arterioles, resulting in thrombosis and ischemia [9]. Among the underlying mechanisms of SCR, inflammation plays a significant role and the inflammatory responses stimulate endothelial cells and immune cells. Ultimately, the production of inflammatory molecules contributes to systemic vaso-occlusion in microvessels [10].

Numerous studies have demonstrated elevated circulating levels of proinflammatory cytokines, such as interleukin (IL)-1β, IL-6, IL-8, and tumor necrosis factor α (TNF-α), in SCD patients during steady-state conditions. These cytokines are associated with chronic endothelial activation, leukocyte aggregation and the potential adhesion of sickle-shaped RBCs, which can lead to ischemia and tissue necrosis [11, 12].

Inflammatory biomarkers, including the neutrophil-to-lymphocyte ratio (NLR), platelet-to-lymphocyte ratio (PLR), systemic immune-inflammation index (SIII: (Neutrophils×Platelets)/Lymphocytes), and systemic inflammation response index (SIRI: (Neutrophils×Monocytes)/Lymphocytes) reflect the overall inflammatory status and immune response. SIII, a novel biomarker, has implications in malignancies and inflammatory diseases. Meanwhile, SIRI provides insight into the balance between inflammatory responses and immune status [13–15].

Recent investigations have explored the predictive value of these indices for various ocular diseases, including dry eye syndrome, retinal vein occlusion, neovascular glaucoma, diabetic retinopathy, and age-related macular degeneration [16–19].

The aim of this study was to determine the relationship between peripheral blood inflammatory indexes such as NLR, PLR, SIII, SIRI, lymphocyte-monocyte ratio (LMR), aggregate index of systemic inflammation (AISI: (Neutrophils×Monocytes×Platelets)/Lymphocytes) and a new parameter defined by us, systemic immune-modulation index (SIMI: (Monocytes×Platelets)/Lymphocytes), with SCD and SCR.

## Methods

### Data collection

The demographic data of all participants were recorded. Haemoglobin, white blood cell (WBC), neutrophil, lymphocyte, monocyte and platelet counts were obtained from a complete blood count (CBC) measurement. Additionally, peripheral blood sample measurements were performed. Hemoglobin electrophoresis was performed for all participants. The NLR, PLR, LMR, SIII, SIRI, AISI, and SIMI were calculated for all participants. All measurements were performed on the same day as the ophthalmologic examination. (Table 1)

**Table 1:** Description of parameters and their units

	Description	Units
NLR	Neutrophil-to-Lymphocyte Ratio	unitless
	Neutrophils count divided by lymphocytes count	
PLR	Platelet-to-Lymphocyte Ratio	unitless
	Platelets count divided by lymphocytes count	
LMR	Lymphocyte-to-Monocytes Ratio	unitless
	Lymphocytes count divided by monocytes count	
SIII	Systemic immune inflammation index	109/L
	(Neutrophils × Platelets) / Lymphocytes	
SIRI	Systemic inflammation response index	109/L
	(Neutrophils × Monocytes) / Lymphocytes	
AISI	Aggregate index of systemic inflammation	1018/L
	(Neutrophils × Monocytes × Platelets) / Lymphocytes	
SIMI	Systemic inflammation modulation index	109/L
	(Monocytes × Platelets) / Lymphocytes	

### Inclusion and exclusion criteria

Patients with a history of diseases that affect inflammatory biomarkers such as diabetes mellitus, cardiovascular disease, systemic arterial hypertension, chronic obstructive pulmonary disease, thyroid disorders, malignancies, renal dysfunction and liver dysfunction, were excluded. Patients with a history of chronic systemic inflammatory connective tissue disease or previous intraocular surgery, ocular inflammation, uveitis, keratoconus, age-related macular degeneration, retinal occlusive disease or glaucoma were also excluded. In addition, patients who had received colony-stimulating factor (CSF) and/or anti-inflammatory treatment for any reason within the last six months were not included.

### Study groups

All the participants included in this study were categorized into distinct groups. Healthy controls constituted group 1 (*n* = 45), while individuals with sickle cell disease (SCD) comprised group 2 (*n* = 100). Among those with SCD (group 2), further subdivisions were made: group 3 (*n* = 74) consisted of SCD patients without SCR, whereas group 4 (*n* = 26) included those with SCR. Withn the latter group (group 4), patients were further classified into group 5 (non-proliferative sickle cell retinopathy (NPSCR), *n* = 12) and group 6 (proliferative sickle cell retinopathy (PSCR), *n* = 14).

Diagnosis and staging of sickle cell retinopathy.

The presence of SCR was defined by the occurrence of any of the following findings: black sunburst lesions, “salmon patch” hemorrhages in the retinal periphery, arteriovenous anastomoses, vascular tortuosity, central retinal artery or vein occlusions, peripheral “seafan” retinal neovascularization, macular hemorrhage, neovascularization of the optic disc, vitreous hemorrhage, or tractional retinal detachment. Goldberg classification was used to classify patients with proliferative sickle cell retinopathy. Color fundus photography was used for diagnosis and staging. All evaluations were performed by experts with at least 5 years of experience. Three physicians evaluated each patient.

### Statistical analysis

Continuous data are presented as the mean ± standard deviation. The Shapiro-Wilk test was used to assess the normality of the distribution. The Student’s t-test was used to compare the means of two independent groups, and one-way ANOVA was used to compare the means of more than two groups. Categorical data are presented as numbers and percentages, and the chi-square test was used for comparison. The area under the curve (AUC) receiver operating characteristic (ROC) was calculated from numerical data. The cutoff value, sensitivity, and specificity of each parameter were also determined. All analyses were performed using MedCalc v.22.018 (MedCalc Software; Ostend, Belgium). The statistical significance level was set at *p* < 0.05. Bonferroni correction was used when comparing patient subgroups. The statistical significance level in subgroup comparison was determined as *p* < 0.008.

## Results

The mean age was 36.8 ± 13.1 years in Group 1 (*n* = 45) and 37.7 ± 10.8 years in Group 2 (*n* = 100). Among Group 1 participants, 20 (44.4%) are female, while Group 2 comprises 46 (46%) female patients. No significant differences were observed between the groups in terms of age and gender (*p* = 0.724 and *p* = 0.863, respectively).

In haemoglobin parameters, the mean haemoglobin level in Group 1 was 14.5 ± 2.36 g/dL, while the mean haemoglobin level in Group 2 was 8.22 ± 1.64 g/dL. The mean HbA level in Group 1 was 97.2%, and the mean HbS level in Group 2 was 63.3%. There was a significant difference between the groups in terms of hemoglobin parameters (*p* < 0.001). In addition, patients were divided into homozygotes and heterozygotes. All parameters were compared. However, no significant difference was found between homozygotes and heterozygotes. Therefore, no further analysis was performed (Table 2).

**Table 2:** Demographic data and hemoglobin electrophoresis results

	Control		SCD		*p*	SCD-woR		SCR		*p*	NPSCR		PSCR		*p*
	Group 1		Group 2			Group 3		Group 4			Group 5		Group 6		
*N*	45		100			74		26			12		14		
	Mean	SD	Mean	SD		Mean	SD	Mean	SD		Mean	SD	Mean	SD	
Age (years)	36.8	13.1	37.74	10.83	0.724	37.00	10.33	39.85	12.13	0.141	44.00	12.24	36.29	11.25	0.936
Hb (g/dL)	14.5	2.36	8.22	1.64	< 0.001	8.27	1.75	8.07	1.29	0.490	8.37	1.20	7.81	1.35	0.290
	n	%	n	%	p	n	%	n	%	p	n	%	n	%	p
Male (n,%)	25	55.60	54	54.00	0.863	38	51.40	16	61.50	0.370	7	58.30	9	64.30	0.757
Female (n,%)	20	44.40	46	46.00		36	48.60	10	38.50		5	41.70	5	35.70	
HbA (%)	97.2	3.3	22.34	25.90	< 0.001	21.49	26.07	24.75	25.78	0.817	29.93	29.55	20.31	22.20	0.068
HbA2 (%)	2.6	1.1	3.91	0.97		3.92	0.89	3.88	1.18	0.091	3.62	1.25	4.10	1.11	0.757
HbF (%)	0	0	9.91	6.89		10.65	7.27	7.79	5.25	0.067	7.78	6.05	7.80	4.70	0.391
HbS (%)	0	0	63.32	22.77		63.49	22.80	62.84	23.13	0.823	57.63	26.12	67.31	20.12	0.119

In the complete blood count, Group 1 exhibits the following mean values: WBC count is 7.17 ± 1.44 × 10^9^/L, neutrophil count is 4.07 ± 1.20 × 10^9^/L, lymphocyte count is 2.30 ± 0.62 × 10^9^/L, monocyte count is 0.55 ± 0.13 × 10^9^/L and platelet count is 259.04 ± 53.12 × 10^9^/L. In Group 2, the corresponding values are as follows: WBC count is 11.00 ± 5.09 × 10^9^/L, neutrophil count is 6.41 ± 3.96 × 10^9^/L, lymphocyte count is 3.06 ± 1.51 × 10^9^/L, monocyte count is 1.13 ± 0.63 × 10^9^/L and platelet count is 382.9 ± 163.8 × 10^9^/L. There was a statistically significant difference in all parameters between the groups. All parameters were higher in Group 2 compared to Group 1 (*p* < 0.001, for all) (Table 3).

**Table 3:** Level of inflammatory biomarkers in peripheral blood

	Control		SCD		*p*	SCD-woR		SCR		*p*	NPSCR		PSCR		*p*
	Group 1		Group 2			Group 3		Group 4			Group 5		Group 6		
*N*	45		100			74		26			12		14		
	Mean	SD	Mean	SD		Mean	SD	Mean	SD		Mean	SD	Mean	SD	
White blood cells (109/L)	7.17	1.44	11.00	5.09	< 0.001	10.19	4.82	13.30	5.22	0.473	12.32	3.95	14.14	6.12	0.109
Neutrophils (109/L)	4.07	1.20	6.41	3.96	< 0.001	5.69	3.59	8.45	4.31	0.169	7.49	3.22	9.27	5.03	0.093
Lymphocytes (109/L)	2.30	0.62	3.06	1.51	< 0.001	3.08	1.50	3.01	1.57	0.835	3.17	1.92	2.87	1.26	0.169
Monocytes (109/L)	0.55	0.13	1.13	0.63	< 0.001	1.02	0.60	1.44	0.63	0.932	1.24	0.55	1.61	0.66	0.511
Platelets (109/L)	259.04	53.12	382.89	163.83	< 0.001	357.65	156.25	454.73	166.57	0.498	408.50	187.92	494.36	140.76	0.423
NLR	1.89	0.73	2.91	4.04	0.011	2.53	3.66	3.98	4.90	0.209	4.33	6.96	3.69	2.22	0.264
PLR	119.11	35.06	152.83	102.78	0.001	139.66	94.70	190.32	116.91	0.064	174.18	132.27	204.15	105.04	0.907
LMR	4.40	1.49	3.22	1.94	0.343	3.57	2.06	2.20	1.01	0.057	2.58	1.11	1.88	0.82	0.473
SIII (109/L)	489.86	209.37	1049.35	1516.01	0.004	819.46	1419.78	1703.67	1616.63	0.043	1532.09	2075.17	1850.74	1153.96	0.555
SIRI (109/L)	1.04	0.53	2.94	3.06	< 0.001	2.35	2.83	4.61	3.12	0.146	3.20	1.46	5.82	3.68	0.023
AISI (1018/L)	270.51	144.18	1113.75	1309.25	< 0.001	734.03	802.87	2194.49	1806.72	< 0.001	1271.45	809.88	2985.66	2064.41	0.011
SIMI (109/L)	63.92	20.44	149.26	96.62	< 0.001	117.31	59.04	240.20	123.15	< 0.001	168.20	71.74	301.92	126.19	0.016

In inflammatory biomarkers, the mean NLR in Group 1 was 1.89 ± 0.73 and the mean PLR was 119.11 ± 35.07. The mean NLR in Group 2 was 2.91 ± 4.04, and the mean PLR was 152.83 ± 102.78. There was a statistically significant difference in NLR and PLR between the groups. NLR and PLR were higher in Group 2 compared to Group 1 (*p* = 0.011, *p* = 0.001, respectively) (Table 3).

In Group 1, the mean values for SIII, SIRI, AISI, and SIMI are 489.86 ± 209.37 × 10^9^/L, 1.04 ± 0.53 × 10^9^/L, 270.51 ± 144.18 × 10^18^/L and 63.92 ± 20.44 × 10^9^/L respectively. In Group 2, the corresponding values are 1049.35 ± 1516.01 × 10^9^/L, 2.94 ± 3.06 × 10^9^/L, 1113.75 ± 1309.25 × 10^18^/L and 149.26 ± 96.62 × 10^9^/L respectively. Statistically significant differences exist between the groups for SIII, SIRI, AISI, and SIMI, with all parameters being higher in Group 2 compared to Group 1 (*p* = 0.004 for SIII, *p* < 0.001 for remains). (Table 3)

In Group 3, the mean AISI is 734 ± 802.9 × 10^18^/L and SIMI is 117.3 ± 59.04 × 10^9^/L. In Group 4, the corresponding values are 2194.5 ± 1806.7 × 10^18^/L and 240.2 ± 123.2 × 10^9^/L respectively. Statistically significant differences exist between the groups for AISI and SIMI with all parameters being higher in Group 4 compared to Group 3 (*p* < 0.001 for both).

In Group 5, the mean SIRI is 3.20 ± 1.46 × 10^9^/L, AISI is 1271.5 ± 809.9 × 10^18^/L and SIMI is 168.20 ± 71.74 × 10^9^/L. In Group 6, the corresponding values are 5.82 ± 3.68 × 10^9^/L, 2985.7 ± 2064.4 × 10^18^/L and 301.9 ± 126.2 × 10^9^/L respectively. There were differences in SIRI, AISI and SIMI between the groups and all parameters were higher in Group 6 compared to Group 5. However, these differences were not statistically significant (*p* = 0.023, *p* = 0.011 and *p* = 0.016, respectively) (Table 3).

**Fig. 1:**
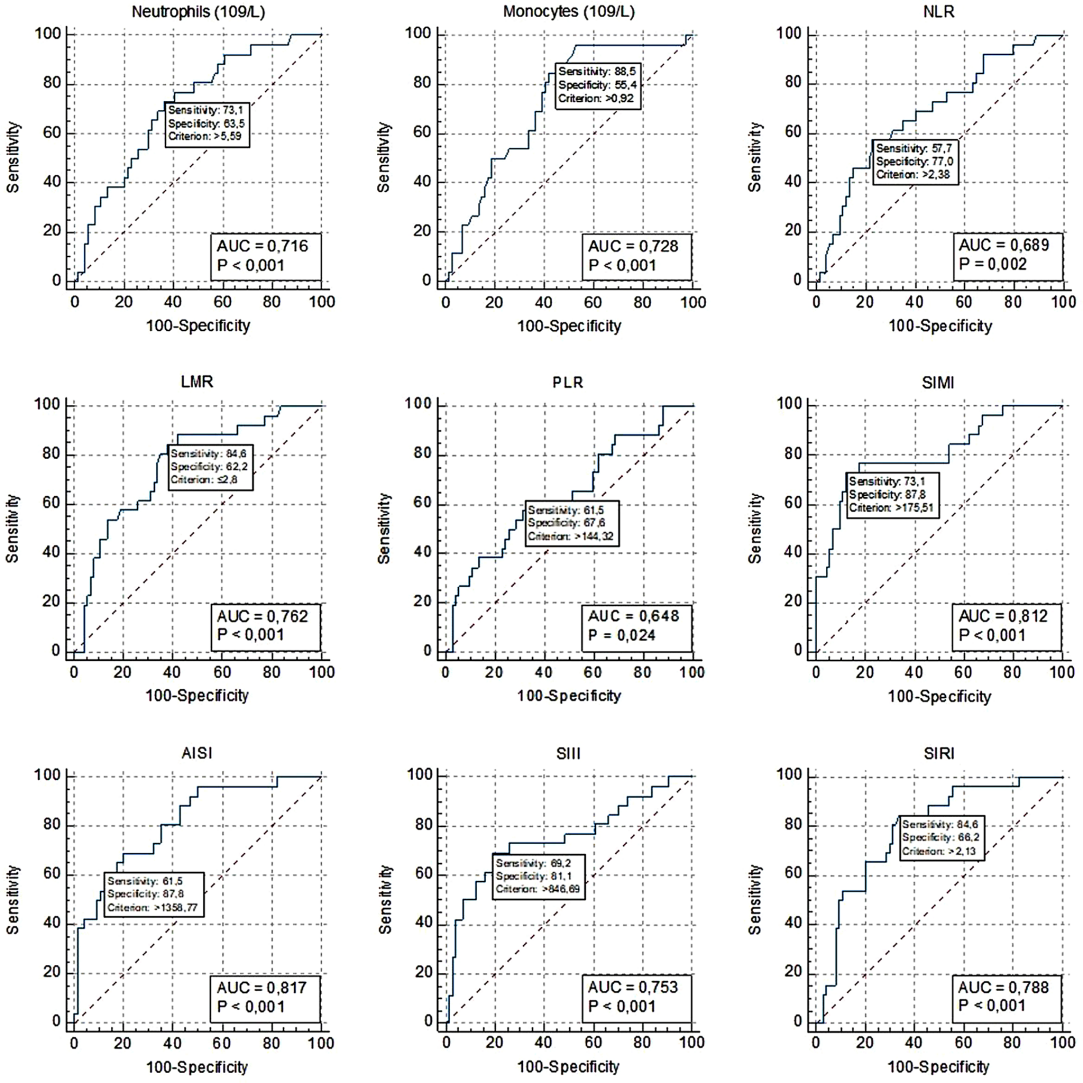
Results of ROC analysis to distinguish patients with SCR (Group 4) from those without retinopathy (Group 3)

To distinguish patients with SCR (Group 4) from those without (Group 3), ROC analysis was performed (Fig. 1). In these analyses, the white blood cell count, neutrophil count and monocyte count parameters demonstrated statistically significant discriminatory power (*p* = 0.001, *p* < 0.001 and *p* < 0.001 respectively). The corresponding areas under the curve (AUCs) were 0.693 [0.59–0.78], 0.716 [0.62–0.80] and 0.728 [0.63–0.81]. The cutoff values were 11.38 × 10^9^/L, 5.59 × 10^9^/L and 0.92 × 10^9^/L respectively. The NLR and LMR parameters also exhibited statistically significant discriminatory power (*p* = 0.002 and *p* < 0.001 respectively). The corresponding AUCs were 0.689 [0.59–0.78] and 0.762 [0.67–0.84]. The cutoff values were 2.38 and 2.8 respectively. Furthermore, the SIII, SIRI, AISI, and SIMI parameters demonstrated significant discriminatory power (*p* = 0.0002, *p* < 0.001, *p* < 0.001, and *p* < 0.001, respectively). The corresponding AUCs were 0.753 [0.66–0.83], 0.788 [0.70–0.86], 0.817 [0.73–0.89] and 0.812 [0.72–0.88]. The cutoff values were 846.69 × 10^9^/L, 2.13 × 10^9^/L, 1358.77 × 10^18^/L and 175.51 × 10^9^/L respectively (Table 4).

**Table 4:** Results of ROC analysis to distinguish patients with SCR (Group 4) from those without retinopathy (Group 3)

Parameters	AUC [95%CI]	*p*	Cut off	Sensitivity	95% CI	Specificity	95% CI
White blood cells (109/L)	0.693[0.59–0.78]	**0.0012**	> 11.38	65.38	44.3–82.8	72.97	61.4–82.6
Neutrophils (109/L)	0.716[0.62–0.80]	**< 0.001**	> 5.59	73.08	52.2–88.4	63.51	51.5–74.4
Lymphocytes (109/L)	0.526[0.42–0.63]	0.6984	≤ 1.77	34.62	17.2–55.7	79.73	68.8–88.2
Monocytes (109/L)	0.728[0.63–0.81]	**< 0.001**	> 0.92	88.46	69.8–97.6	55.41	43.4–67.0
Platelets (109/L)	0.663[0.56–0.76]	**0.0092**	> 375	69.23	48.2–85.7	63.51	51.5–74.4
NLR	0.689[0.59–0.78]	**0.0019**	> 2.38	57.69	36.9–76.6	77.03	65.8–86.0
PLR	0.648[0.55–0.74]	**0.0245**	> 144.32	61.54	40.6–79.8	67.57	55.7–78.0
LMR	0.762[0.67–0.84]	**< 0.001**	≤ 2.8	84.62	65.1–95.6	62.16	50.1–73.2
SIII (109/L)	0.753[0.66–0.83]	**0.0002**	> 846.69	69.23	48.2–85.7	81.08	70.3–89.3
SIRI (109/L)	0.788[0.70–0.86]	**< 0.001**	> 2.13	84.62	65.1–95.6	66.22	54.3–76.8
AISI (1018/L)	0.817[0.73–0.89]	**< 0.001**	> 1358.77	61.54	40.6–79.8	87.84	78.2–94.3
SIMI (109/L)	0.812[0.72–0.88]	**< 0.001**	> 175.51	73.08	52.2–88.4	87.84	78.2–94.3

**Fig. 2:**
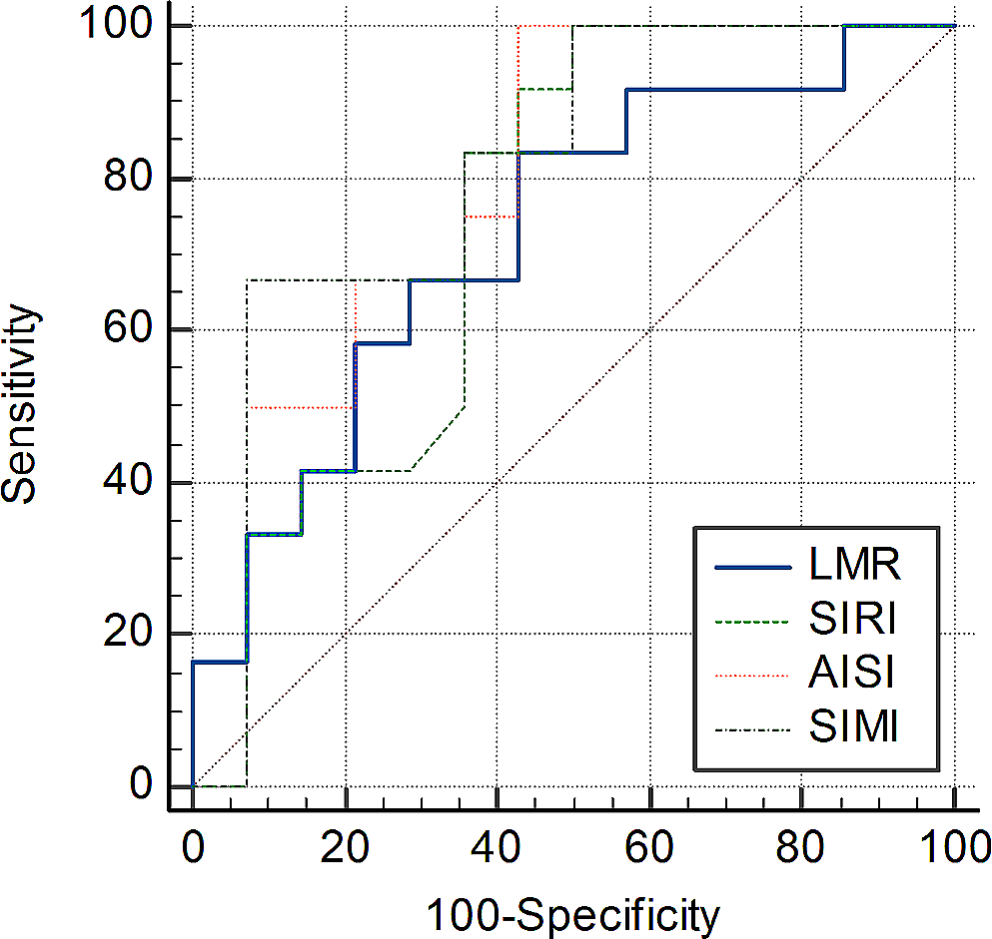
Results of ROC analysis distinguish patients with non-proliferative sickle cell retinopathy (Group 5) from those proliferative sickle cell retinopathy (Group 6) LMR: Lymphocyte-to-Monocytes Ratio, SIRI: Systemic inflammation response index, AISI: Aggregate index of systemic inflammation, SIMI: Systemic inflammation modulation index

To distinguish non-proliferative sickle cell retinopathy (Group 5) from proliferative sickle cell retinopathy (Group 6), ROC analysis was also performed (Fig. 2). In these analyses, AISI and SIMI parameters demonstrated statistically significant discriminatory power (*p* = 0.002 and *p* < 0.001 respectively). The corresponding AUCs were 0.792 [0.59–0.93], and 0.810 [0.61–0.94]. The cutoff values were 2819.51 × 10^18^/L and 185.86 × 10^9^/L, respectively (Table 5).

**Table 5:** Results of ROC analysis to distinguish patients with non-proliferative sickle cell retinopathy (Group 5) from those proliferative sickle cell retinopathy (Group 6)

Parameters	AUC [95%CI]	*p*	Cut off	Sensitivity	95% CI	Specificity	95% CI
White blood cells (109/L)	0.545[0.40–0.74]	0.7093	> 18.48	28.57	8.4–58.1	100	73.5–100.0
Neutrophils (109/L)	0.595[0.39–0.78]	0.4124	> 9.51	42.86	17.7–71.1	83.33	51.6–97.9
Lymphocytes (109/L)	0.524[0.32–0.72]	0.845	≤ 3.81	85.71	57.2–98.2	33.33	9.9–65.1
Monocytes (109/L)	0.685[0.47–0.85]	0.0884	> 1.08	85.71	57.2–98.2	50	21.1–78.9
Platelets (109/L)	0.667[0.46–0.84]	0.154	> 437	64.29	35.1–87.2	75	42.8–94.5
NLR	0.613[0.40–0.80]	0.3307	> 4.17	35.71	12.8–64.9	91.67	61.5–99.8
PLR	0.637[0.43–0.82]	0.2424	> 101	92.86	66.1–99.8	41.67	15.2–72.3
LMR	0.726[0.52–0.88]	**0.0276**	≤ 1.56	57.14	28.9–82.3	83.33	51.6–97.9
SIII (109/L)	0.685[0.47–0.85]	0.107	> 856.29	85.71	57.2–98.2	58.33	27.7–84.8
SIRI (109/L)	0.741[0.53–0.89]	**0.0185**	> 5.56	50	23.0–77.0	100	73.5–100.0
AISI (1018/L)	0.792[0.59–0.93]	**0.0016**	> 2819.51	57.14	28.9–82.3	100	73.5–100.0
SIMI (109/L)	0.810[0.61–0.94]	**0.0006**	> 185.86	92.86	66.1–99.8	66.67	34.9–90.1

In univariate analysis, PLT (*p* = 0.037) and SIMI (*p* = 0.001) were significantly associated with the severity of sickle cell retinopathy (proliferative and non-proliferative), and only SIMI (*p* = 0.005) was significantly associated with the development of sickle cell retinopathy. In multivariate analysis, only SIMI was associated with development and severity of sickle cell retinopathy (*p* = 0.001).

## Discussion

Sickle cell disease is the most common genetic hematological disorder with an estimated 300,000 affected births annually worldwide [20]. Unfortunately, many investigated biomarkers have limitations that have not supported their adoption into clinical practice, such as modest diagnostic and prognostic accuracy, long turn around time and high cost. A complete blood count is a valuable test that provides rich information about an individual’s health status. This test has several advantages, including being inexpensive, easy to perform and widespread availability across various healthcare services [21].

In recent years, many studies have been published using novel biomarkers that can be easily calculated with blood parameters to determine systemic inflammation. Understanding the pathogenesis of diseases contributes to the identification of new therapeutic targets. On the other hand, it can be a guide in monitoring and prognosis determination. Overall, there are no studies on the relationship between SCD and SCR with the inflammatory biomarkers (NLR, PLR, SII, SIRI, AISI, and SIMI) used in our study. Therefore, we believe that our study could be a pioneering effort in this regard.

In a study conducted on 262 participants with SCD, the median age of participants was 20 years. The prevalence of NPSCR in this cohort was 24%. Approximately 2% of patients exhibited PSCR. Independent predictors of retinopathy included elevated systolic blood pressure, moderate visual impairment and anterior segment changes [22]. Another study investigated risk factors associated with the development of SCR and included 50 SCD patients. The results revealed an association between increased E-selectin levels and SCR [23]. Endothelial activation, triggered by inflammatory stimuli such as interleukin-1β (IL1-β) and tumor necrosis factor-α (TNF-α), disrupts nitric oxide (NO) homeostasis and modulates the expression of key adhesion molecules [24]. Among these molecules, P-selectin, E-selectin, intercellular adhesion molecule-1 (ICAM-1) and vascular cell adhesion molecule-1 (VCAM-1) play pivotal roles in the vaso-occlusive process and potentially contribute to development of SCR [25]. Notably, low-density circulating reticulocytes and leukocytes serve as ligands for these adhesion molecules, including the VLA-4 integrin [25, 26]. Furthermore, sickle cell retinas obtained from postmortem ocular tissue exhibit an elevated abundance of polymorphonuclear leukocytes compared to healthy [26]. Based on this data, we also attempted to present the changes in blood-cell associated inflammation parameters in the presence of SCD and SCR.

A study involving 37 patients with SCR, 34 SCD patients without retinopathy, and healthy found significantly lower soluble intercellular adhesion molecule-1 (sICAM-1) levels and higher pigment epithelium-derived factor (PEDF) levels in SCR patients. Furthermore, SCD patients exhibited elevated levels of angiopoietin-1 (Ang-1), angiopoietin-2 (Ang-2), and interleukin-1β (IL1-β) compared to healthy [27]. In our study, blood-cell associated inflammation parameters were examined in patients with SCD and SCR. SCD patients had significantly higher neutrophil, lymphocyte, monocyte and platelet counts compared to healthy. Additionally, NLR, PLR, SIII, SIRI, AISI and SIMI results were also statistically significantly higher in SCD patients.

Lard et al. presented that white blood cells, particularly neutrophils, may play a role in the onset and progression of vaso-occlusive events [28]. The adhesion of activated neutrophils to the endothelium in SCD can result in endothelial damage, contributing to blood flow obstruction in the microcirculation due to the resilience of neutrophils compared to red blood cells [29]. Furthermore, the recruitment of adherent leukocytes to activated endothelium exacerbates the progression vascular complications [30]. The presence of activated platelets, which are common in SCA patients, may worsen vaso-occlusion [30]. These patients exhibit elevated platelet counts and enhanced platelet activation even during steady state, with platelet counts rising further during vaso-occlusive events [31, 32]. Vascular endothelial abnormalities are pivotal in the development of end-organ diseases. Recently, the NLR and PLR have emerged as biological markers of subclinical inflammation, with elevated levels linked to adverse clinical outcomes in cardiovascular diseases, cancers and renal and gastrointestinal disorders, which are linked with SCD [33–36]. In the present study, neutrophil, leukocyte, and platelet counts, as well as NLR and PLR values, were higher in patients with SCD.

In recent years, SIII and SIRI have emerged as novel inflammatory biomarkers [37]. Studies have demonstrated that SIII and SIRI incorporate platelets and various inflammatory cells among white blood cells encompassing a range of immune regulatory pathways within the human body. Compared to the analysis of white blood cells or platelets, SIII and SIRI exhibit enhanced stability across diverse physiological and pathological states. This characteristic allows them to offer a more robust reflection of the organism’s overall inflammatory status [38]. Specifically, elevated SIII levels have been indicated as a potential risk factor for ischemic retinal pathologies such as diabetic retinopathy, age related macular degeneration, and retinopathy of prematurity [39, 40]. In addition, Wang et al. demonstrated that SIRI, in combination with SIII, can also serve as an independent risk factor for the development of diabetic retinopathy [37]. In our study, SIII and SIRI have shown significant results in predicting the development of retinopathy in SCD patients. Additionally, SIRI has been shown to be one of the inflammatory parameters that indicates progression to proliferation in patients with SCR.

The aggregate index of systemic inflammation (AISI), distinct from other inflammation indexes based on hematological parameters, integrates data from four key blood cell types implicated in inflammation: neutrophils, monocytes, platelets, and lymphocytes. Initially explored in 2018 for predicting outcomes in surgical patients [41]. AISI has since garnered attention for its potential clinical relevance in various disease contexts characterized by systemic pro-inflammatory states such as age related macular degeneration, cancer and idiopathic pulmonary fibrosis [42–45]. Particularly noteworthy is the finding in idiopathic pulmonary fibrosis patients, where the AISI exhibited superior prognostic capacity for four-year survival compared to individual neutrophils, monocytes, lymphocytes, and platelets. This advantage also extended to established inflammatory indexes, such as the NLR and PLR [45]. According to our results, AISI was significantly higher in SCD patients, and it seems that can help us to distinguish patients with SCR from non-retinopathic SCD patients as well as PSCR patients from NPSCR patients.

Monocytes play a multifaceted role in tissue repair, pathogens elimination, and the initiation of adaptive immune responses. However, when recruited, monocytes can also contribute to the pathogenesis of infectious diseases and chronic inflammatory conditions, such as atherosclerosis [46]. Additionally, platelets exhibit the capacity to release various mediators, including thromboxane, which can contribute to heightened inflammation [47]. Elevated platelet counts often trigger increased thrombocyte activation, a process pivotal for megakaryocytic proliferation and subsequent thrombocytosis. Furthermore, augmented platelet activation significantly influences the initiation and progression of inflammation and atherosclerosis [47]. SIMI is the ratio of (monocyteXplatelet count) to lymphocytes, and according to our results, a usable parameter to distinguish the presence of retinopathy and the severity of retinopathy in SCD patients. The proposed SIMI reflects the potential for platelet-mediated modulation of monocyte function. Platelets are known to influence monocyte differentiation and activity [48]. They promote a shift towards pro-inflammatory CD16 + monocyte subsets (non-classical and intermediate) through interaction with CD16 receptors [48]. Additionally, platelet-derived signals directly and indirectly regulate the expression of pro-inflammatory cytokines by monocytes, including MCP-1, IL-1β, IL-6, IL-8, IL-12, and MIP-1β [49]. This suggests that SIMI could potentially serve as a biomarker for inflammatory processes mediated by platelet-monocyte interactions.

This study has some limitations. The study included a considerable number of SCD patients. However, when these patients were divided into subgroups according to the presence and severity of retinopathy, the resulting subgroups had relatively few participants. Consequently, despite obtaining higher values in some inflammation parameters in the PSCR group compared to the NPSCR group, no statistically significant difference was found between the groups. Furthermore, due to the cross-sectional design of this study, the patients’ status during a steady state or acute crisis was not investigated. The strengths of our study include the fact that it is the first study to investigate systemic immune indexes in patients with SCR and the first study with a newly defined index obtained from patients with SCD, the systemic inflammation modulation index (SIMI).

In conclusion, blood-cell associated inflammation parameters (NLR, PLR, SIII, SIRI, AISI, and SIMI) were statistically higher in patients with SCD, and all of these parameters had significant discriminatory power in SCD patients with and without retinopathy. In addition to the presence of diabetic retinopathy, SIRI, AISI, and SIMI also revealed significant results in discriminating the severity of retinopathy in SCR patients. Considering the importance of inflammatory processes in the development of SCD and its potential complications, the evaluation of blood-cell-associated inflammation markers in SCD seems to be quite an easy and useful method in patient management and treatment.
